# Synergistic Cytotoxic Effect of L-Asparaginase Combined with Decitabine as a Demethylating Agent in Pediatric T-ALL, with Specific Epigenetic Signature

**DOI:** 10.1155/2016/1985750

**Published:** 2016-11-27

**Authors:** Salvatore Serravalle, Salvatore N. Bertuccio, Annalisa Astolfi, Fraia Melchionda, Andrea Pession

**Affiliations:** ^1^Pediatric Hematology and Oncology, Department of Pediatrics, S.Orsola-Malpighi Hospital, University of Bologna, Bologna, Italy; ^2^“Giorgio Prodi” Cancer Research Center, University of Bologna, Bologna, Italy

## Abstract

T-Acute Lymphoblastic Leukemia (T-ALL) remains a subgroup of pediatric ALL, with a lower response to standard chemotherapy. Some recent studies established the fundamental role of epigenetic aberrations such as DNA hypermethylation, to influence patients' outcome and response to chemotherapy. Moreover, L-asparaginase is an important chemotherapeutic agent for treatment of ALL and resistance to this drug has been linked to* ASNS* expression, which can be silenced through methylation. Therefore, we tested whether the sensitivity of T-ALL cell lines towards L-asparaginase is correlated to the epigenetic status of* ASNS* gene and whether the sensitivity can be modified by concurrent demethylating treatment. Hence we treated different T-ALL cell lines with L-asparaginase and correlated different responses to the treatment with* ASNS* expression. Then we demonstrated that the* ASNS* expression was dependent on the methylation status of the promoter. Finally we showed that, despite the demethylating effect on the* ASNS* gene expression, the combined treatment with the demethylating agent Decitabine could synergistically improve the L-asparaginase sensitivity in those T-ALL cell lines characterized by hypermethylation of the* ASNS* gene. In conclusion, this preclinical study identified an unexpected synergistic activity of L-asparaginase and Decitabine in the subgroup of T-ALL with low* ASNS* expression due to hypermethylation of the* ASNS* promoter, while it did not restore sensitivity in the resistant cell lines characterized by higher* ASNS* expression.

## 1. Introduction

T-Acute Lymphoblastic Leukemia is an aggressive hematologic neoplasm which arises from the malignant transformation of T-cell progenitors and accounts for about 15% of pediatric ALL. Despite the encouraging results of modern chemotherapy ALL protocols, T-ALL remains, also in the pediatric setting, a subgroup with the worst prognosis [1, 2]. T-ALL can be divided into molecular genetic subgroups which are characterized by unique gene expression signatures and aberrant activation of specific T-ALL transcription factor oncogenes, including MEF2C, HOXA, TLX1, NKX2.1, TLX3, TAL1, LMO1, and LMO2, and of oncogenic signaling cascades, including interleukin 7 receptor (IL7R), Janus kinase (JAK), signal transducer and activator of transcription (STAT) [3], phosphatidylinositol 3-kinase (PI3K)/Akt [4], and Ras/mitogen-extracellular signal-regulated kinase (MEK)/extracellular signal-regulated kinase (ERK) [5]. On the other hand, various studies have shown an important role of epigenetic aberrations in the pathogenesis of pediatric acute leukemia, including T-ALL, and resistance to chemotherapy [6]. In particular, Borssen et al. recently showed that the amount of CpG island methylation could predict the outcome and response to standard treatment [7].

L-asparaginase (L-Asp) is an important chemotherapeutic agent for the management of acute lymphoblastic leukemia. Resistance to L-Asp therapy may also be linked to high level of* ASNS* expression, which regulates the production of asparagine. Expression of cellular* ASNS* was found low in B-lineage ALL and T-lineage ALL, thus explaining the high sensitivity to L-Asp treatment [8, 9]. In this regard, some studies described that the low expression of* ASNS* in certain subgroups of ALL is due to the hypermethylation of the* ASNS* promoter [10]. Nevertheless, the correlation of* ASNS* expression and sensitivity to L-Asp is actually discussed and highly controversial [11, 12].

Considering the strong correlation between ALL outcome and epigenetic aberrations such as DNA methylation, the aim of our study was to investigate a possible role of this epigenetic alteration in the sensitivity of T-ALL to L-Asp and explore the efficacy of a combined treatment with demethylating agents that could modulate the sensitivity to L-Asp in this setting.

## 2. Materials and Methods

### 2.1. Cell Culture and Reagents

Human T-ALL cell lines, DND41, HPB-ALL, RPMI 8402, CCRF-CEM, and Jurkat, were grown in RPMI 1640 (Lonza), supplemented with 10% Fetal Bovine Serum (Invitrogen), 2 mM L-glutamine (GIBCO), 100 U/mL penicillin, and 100 *μ*g/mL streptomycin (GIBCO).

### 2.2. Treatment with L-Asparaginase and Decitabine

To test the effects of L-Asp, cell lines were cultured for 48 h in the presence of increasing drug concentrations from 10^−5^ U/mL to 10^2^ U/mL.

To explore the efficacy of a combined treatment with demethylating agents, T-ALL cell lines were cultured and treated with 1 *μ*M of Decitabine for 24 h. After 24 h cell lines were treated with increasing concentrations of L-Asp (from 10^−5^ to 10^2^) for 48 h. Due to high instability, Decitabine was added every 24 h.

L-asparaginase (Erwinase) was purchased from Jazz pharmaceutic; Decitabine instead was purchased from Sigma-Aldrich.

### 2.3. Cell Viability Assay

Cell viability was determined using the WST1 (4-[3-(4-lodophenyl)-2-(4-nitrophenyl)-2H-5-tetrazolio]-1,3-benzene disulfonate) cell proliferation kit (Roche Applied Science, Monza, Italy), according to manufacturer's instructions. Assays were performed in triplicate. Nonlinear regression curve fit and IC50 analysis were calculated using the GraphPad Prism software (San Diego, CA, USA). Statistical analysis was performed using Student's *t*-test at a significance level of *p* < 0.05 (GraphPad Prism Software). For drug-combination experiments, a combination index (CI) was calculated using the Compusyn software. CI values <1 indicate synergism; CI = 1 indicates an additive effect, whereas values > 1 indicate antagonism.

### 2.4. RNA Extraction and Quantitative PCR (qRT-PCR)

Total RNA was extracted by the RNeasy spin column method (Qiagen). 1 *μ*g total RNA was reversely transcribed to single-stranded cDNA using the Transcriptor first strand cDNA synthesis kit (Roche Diagnostics) with oligo-dT primers (2.5 *μ*M).* ASNS* gene-specific primers were designed with Primer Express 3.0 Software (Applied Biosystems, Monza, Italy) and qRT-PCR was performed using FastStart Sybr Green (Roche Diagnostics) on the LightCycler 480 apparatus (Roche Diagnostics). DDCt method was used to quantify gene expression levels, relative to the two housekeeping genes GAPDH and ATP5B. Quantitative RT-PCR primer sequences were as follows: ASNS forward (5′-3′) GACAGAAGGATTGGCTGCCT, ASNS reverse (5′-3′) CATCCAGAGCCTGAATGCCT, GAPDH forward (5′-3′) CCAATATGATTCCACCCATGGC, GAPDH reverse (5′-3′) CTTGATTTTCGAGGGATCTCGC, ATP5B Forward (5′-3′) GTCTTCACAGGTCATATGGGGA, ATP5B reverse (5′-3′)ATGGGTCCCACCATATAGAAGG. In order to prove the anticorrelation between ASNS expression and sensitivity to L-Asp, we calculated *r* ^2^ coefficient with GraphPad Prism software (San Diego, CA, USA). In the same way after 72 h of Decitabine 1 *μ*M treatment, we analyzed the expression of* ASNS* gene in the DND41 cell line.

### 2.5. Methylation Analysis

Genomic DNA was extracted using the QIAamp DNA kit (Qiagen). Methylation analysis was performed using the Epigentek Methylamp modification kit (Epigentek). After bisulfite modification, promoter region of the* ASNS* gene (position, CpG: 74 range = chr7:97871872–97872607) was amplified with specific primers: ASNS forward primer (5′-3′) TTAGGGAATTAGGATAGAAAGGTTT, ASNS reverse primer (5′-3′) AAACAAACCAAATTCAAAAACCTCC 3′. PCR product was purified and labeled with BigDye Terminator 1.1 (Applied Biosystems) and sequenced on a ABI3730 instrument (Applied Biosystems).

## 3. Results

### 3.1. T-ALL Cell Lines Displayed Different Sensitivity to L-Asp Treatment

T-ALL cell lines were cultured with increasing concentrations of L-Asp, ranging from 0,0001 U/mL to 100 U/mL and cell viability was measured 48 hours after treatment.

**Figure 1:**
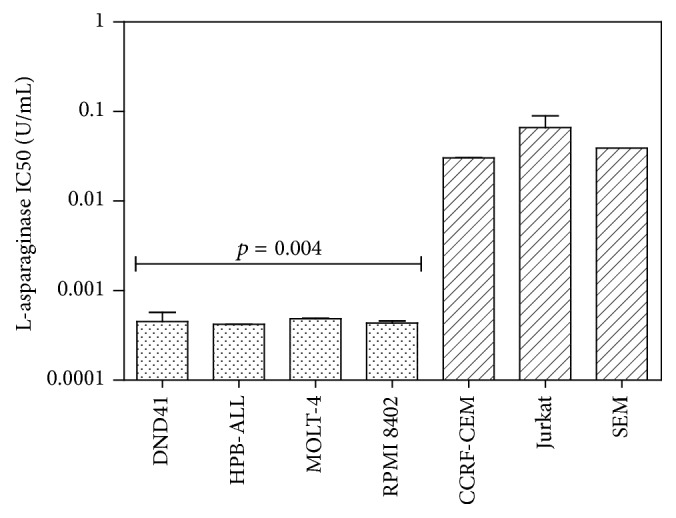
IC50 of L-asparaginase in T-ALL cell lines. DND41, HPB-ALL, and RPMI 8402 displayed higher sensitivity to L-Asp than other T-ALL cell lines (*p* = 0.004).

The value of IC50 in T-ALL cell lines was between 10^−2^ and 10^−4^ U/mL. In particular DND41, HPB-ALL, and RPMI 8402 were more sensitive than other T-ALL cell lines (Jurkat and CCRF-CEM) (*p* = 0.004) (Figure 1).

### 3.2. The Level of L-Asparaginase Sensitivity Correlated with* ASNS* Expression

**Figure 2:**
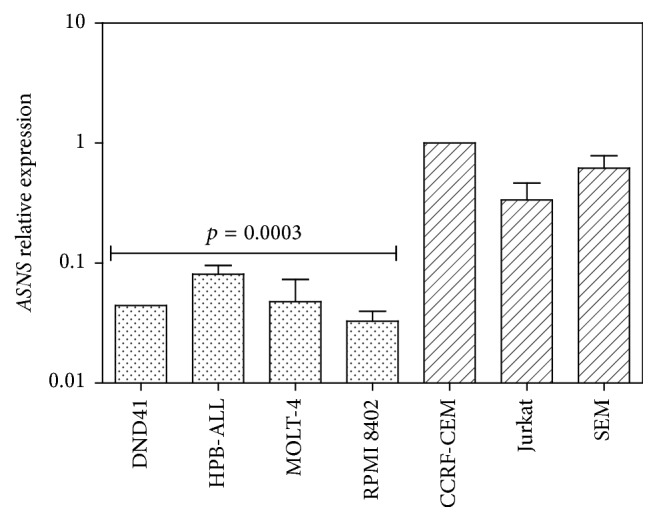
Expression analysis of* ASNS* gene in T-ALL cell lines. Cell lines that were more sensitive to L-Asp had lower expression of the* ASNS* gene than other T-ALL cell lines (*p* = 0.0003).

The expression level of* ASNS* was evaluated by qRT-PCR. The analysis of results showed a variable expression of* ASNS* among T-ALL cell lines. In particular, cell lines displaying high sensitivity to L-Asp treatment had lower expression of the* ASNS* gene, whereas cell lines showing lower sensitivity to L-Asp treatment were characterized by higher expression of* ASNS* gene. Therefore in T-ALL cell lines the sensitivity to L-Asp treatment inversely correlated with the expression level of the* ASNS* gene (*p* = 0.0003), showing a mild positive correlation between the IC50 and the* ASNS* expression, with a *r* ^2^ of 0.6 (Figure 2).

### 3.3. Hypermethylation of the* ASNS* Promoter Correlated with* ASNS* Expression

**Figure 3:**
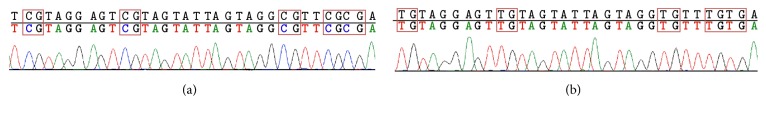
Example of cell lines with (a) hypermethylation of* ASNS* promoter and (b) cell lines without this epigenetic event.

To characterize the mechanism of the* ASNS* gene silencing in T-ALL cell lines, we analyzed the methylation status of the* ASNS* promoter. Sanger sequencing, performed on bisulfite-modified DNA, displayed a complete methylation of CpG islands in* ASNS* promoter in those cell lines which had a low expression of* ASNS* (HPB-ALL, DND41, and RPMI 8402) (Figure 3(a)). On the other hand, Jurkat and CCRF-CEM cells did not display this epigenetic signature and showed a completely unmethylated promoter region (Figure 3(b)).

### 3.4. Decitabine Improved L-Asp Sensitivity in Cell Lines with Hypermethylation of* ASNS* CpG Islands

**Figure 4:**
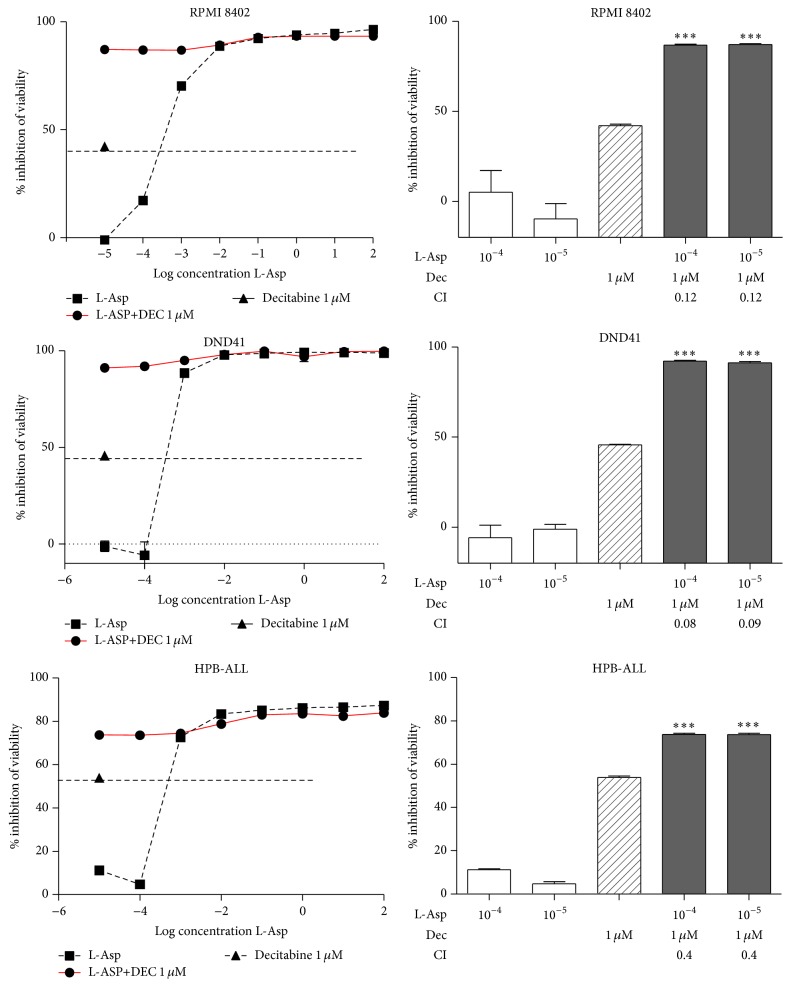
Synergic effect of Decitabine and L-Asp in RPMI 8402, DND41, and HPB-ALL. At 10^−4^ and 10^−5^ U/mL L-Asp alone did not affect cell viability, but addition of 1 *μ*M of Decitabine displayed a statistically significant synergic effect (^*∗∗∗*^ *p* value <0.001). CI values <1 indicates synergism; CI = 1 indicates additive effect, whereas values > 1 indicates antagonism.

**Figure 5:**
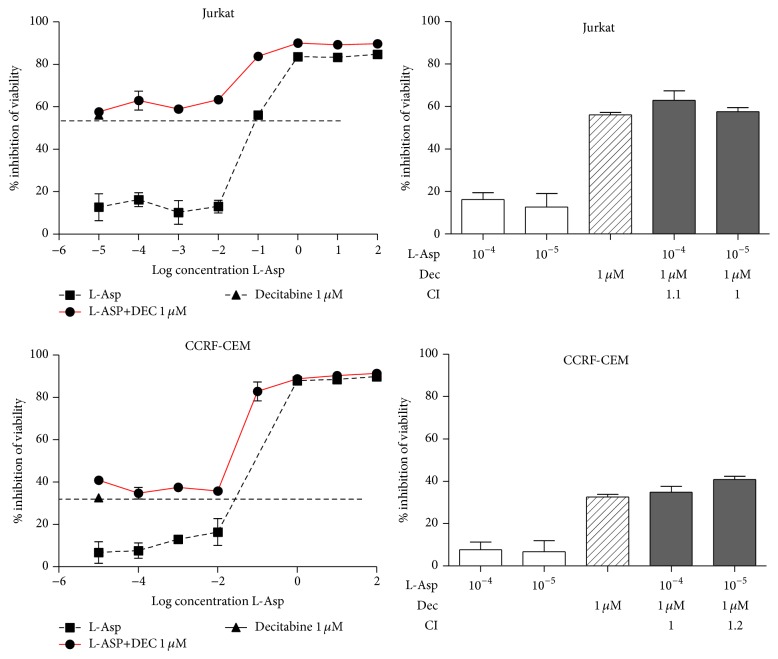
Jurkat and CCRF-CEM did not display synergism between Decitabine and L-asparaginase. CI values <1 indicate synergism; CI = 1 indicates additive effect, whereas values > 1 indicate antagonism.

**Figure 6:**
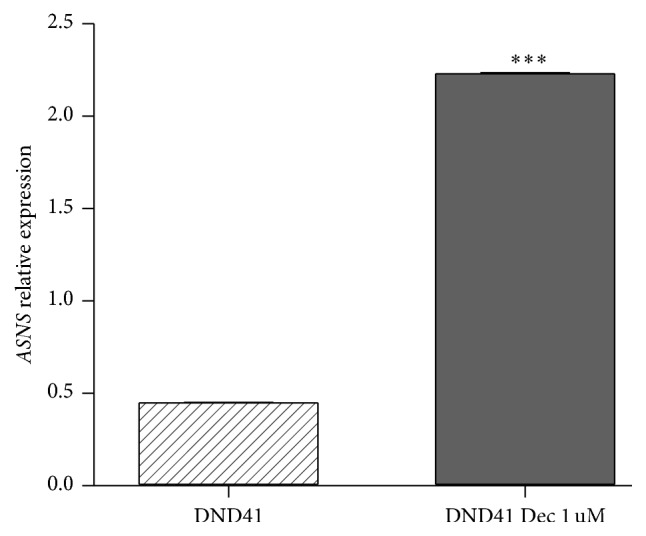
Expression of the* ASNS* gene in DND41 cell line after 72 h of treatment with 1 *μ*M of Decitabine. (^*∗∗∗*^ *p* value <0.001).

To better correlate the* ASNS* expression level with promoter methylation and check the effect of a demethylating agent to modulate L-Asp sensitivity, we treated all T-ALL cell lines with Decitabine 1 *μ*M, in combination with increasing concentrations of L-Asp. Analysis of the results showed a reproducible toxicity in all T-ALL cell lines with about 50% of inhibition of cell viability. Unexpectedly, in cell lines displaying hypermethylation of* ASNS* promoter, Decitabine and L-Asp exhibited a strong synergistic effect with a value of combination index (CI) < 1 (Figure 4). In particular, concentrations of 10^−4^ U/mL and 10^−5^ U/mL of L-Asp alone did not affect cell viability, but the percentage of citotoxicity increased dramatically in association with Decitabine. Other cell lines (Jurkat and CCRF-CEM) without methylation of* ASNS* promoter did not display this combined effect (CI > 1) (Figure 5). To confirm the reactivation of* ASNS* expression after Decitabine treatment, we performed qPCR of* ASNS* gene in DND41 cell line. Following 72 h of treatment with 1 *μ*M of Decitabine, our data showed a significant increase in expression of the* ASNS* gene (*p* < 0.001) (Figure 6).

## 4. Discussion and Conclusion

T-ALL originates from T-cell precursors at different stages of their development and is characterized by distinct and well characterized molecular genetic subtypes. Children affected by this disease respond fairly well to high-dose chemotherapy regimens [1]. L-asparaginase is an important chemotherapeutic agent for the management of acute lymphoblastic leukemia. Resistance to L-Asp therapy may also be linked to high levels of* ASNS* cellular expression, which allows the production of asparagine. Expression of cellular* ASNS* is low in B-lineage ALL, explaining the higher sensitivity to L-Asp treatment. One mechanism implicated in gene silencing of* ASNS* is hypermethylation of the* ASNS* promoter. Moreover, in the last years some studies highlighted that epigenetic aberration may be determinant in inducing resistance to chemotherapy and poor outcome in T-ALL. Treatment with demethylating agents looks promising in an effort to provide new treatment options in T-cell malignant disorders [13]. In this context, our study attempted to investigate an epigenetic marker that could correlate with a high sensitivity to L-Asp and explored the interaction with demethylating agents such as Decitabine. Therefore we treated T-ALL cell lines with increasing concentrations of L-Asp and found a subgroup of T-ALL cell lines displaying a high sensitivity to treatment. Then we confirmed that cell lines showing high sensitivity to L-Asp treatment had a very low expression of* ASNS* gene, which is in line with some literature data [10]. In our study, the mechanism of gene silencing in T-ALL cell lines displaying low expression of* ASNS* gene appeared to be linked to hypermethylation of the* ASNS* promoter as already shown [10]. Unexpectedly, we found a strong synergic effect of L-ASP and Decitabine, a demethylating agent, in cell lines with hypermethylation of* ASNS* at base line evaluation, DND41, HPB-ALL, and RPMI 8402.

This result seems to underline a different role of hypermethylation of* ASNS* promoter in the cellular response to L-Asp.

Therefore other studies are necessary to further explain the molecular mechanism underlying this result, which is probably linked to a larger set of methylated genes in T-ALL subset. In particular, methylation status may not be restricted to* ASNS* gene and the reactivation of many genes such as oncosuppressors could have an important role in the synergic cytotoxic effect induced by Decitabine. The clinical translation of these data is not immediately predictable; however it remains an intriguing result because of the fact that cotreatment with a demethylating agent as Decitabine results in a synergistic effect. The role of demethylating agents in combination with standard chemotherapy should be further analyzed in preclinical and ex vivo models.
